# Longitudinal Studies of Wearables in Patients with Diabetes: Key Issues and Solutions

**DOI:** 10.3390/s23115003

**Published:** 2023-05-23

**Authors:** Ahmad Yaser Alhaddad, Hussein Aly, Hoda Gad, Einas Elgassim, Ibrahim Mohammed, Khaled Baagar, Abdulaziz Al-Ali, Kishor Kumar Sadasivuni, John-John Cabibihan, Rayaz A. Malik

**Affiliations:** 1 Department of Mechanical and Industrial Engineering, Qatar University, Doha 2713, Qatar; ahmadyaser.alhaddad@polimi.it (A.Y.A.); john.cabibihan@qu.edu.qa (J.-J.C.); 2 KINDI Center for Computing Research, Qatar University, Doha 2713, Qatar; ha1601589@qu.edu.qa (H.A.); a.alali@qu.edu.qa (A.A.-A.); 3 Weill Cornell Medicine—Qatar, Doha 24144, Qatar; hyg2002@qatar-med.cornell.edu (H.G.); eie2002@qatar-med.cornell.edu (E.E.); ibm2001@qatar-med.cornell.edu; 4 Department of Internal Medicine, Albany Medical Center Hospital, Albany, NY 12208, USA; 5 Hamad Medical Corporation, Doha 3050, Qatar; kbaagar@hamad.qa; 6 Center for Advanced Materials, Qatar University, Doha 2713, Qatar; kishorkumars@qu.edu.qa

**Keywords:** continuous blood glucose, diabetes management, longitudinal monitoring, wearable devices, data collection, machine learning

## Abstract

Glucose monitoring is key to the management of diabetes mellitus to maintain optimal glucose control whilst avoiding hypoglycemia. Non-invasive continuous glucose monitoring techniques have evolved considerably to replace finger prick testing, but still require sensor insertion. Physiological variables, such as heart rate and pulse pressure, change with blood glucose, especially during hypoglycemia, and could be used to predict hypoglycemia. To validate this approach, clinical studies that contemporaneously acquire physiological and continuous glucose variables are required. In this work, we provide insights from a clinical study undertaken to study the relationship between physiological variables obtained from a number of wearables and glucose levels. The clinical study included three screening tests to assess neuropathy and acquired data using wearable devices from 60 participants for four days. We highlight the challenges and provide recommendations to mitigate issues that may impact the validity of data capture to enable a valid interpretation of the outcomes.

## 1. Introduction

Monitoring of one’s health has never been easier and more convenient thanks to the technological progress made in wearables. These lightweight devices can be interfaced with computers and smartphones to measure and collect different physiological variables such as activity level, heart rate, temperature, and respiration rate through watches, wrist bands, bracelets, lenses, earphones, socks, and glasses [1]. The popularity of wearables has further increased due their engaging users in more active and healthy lifestyles, especially with the adoption of smartwatches and fitness trackers [2,3]. Wearable devices have been increasingly integrated into healthcare delivery to detect falls among the elderly, characterize interactions with social robots, and diagnose children with autism [4,5,6,7,8,9,10,11].

In 2021, the International Diabetes Federation estimated that there were 537 million people with diabetes and predicted that this number will reach 700 million by 2045. Patients with diabetes need to maintain optimal blood glucose levels and avoid hypoglycemia by monitoring their blood sugar, regulating their calorie intake, and adhering to treatment strategies [12,13,14,15]. Hyperglycemia and hypoglycemia can adversely affect quality of life and lead to acute and long-term complications [16,17,18]. Blood glucose measurement most commonly relies on a finger prick with a drop of blood on a disposable test strip, which can cause discomfort, pain, and scarring [19]. Continuous glucose monitoring (CGM) is a minimally invasive technology that measures interstitial glucose with a time lag with blood glucose and varying degrees of reliability, accuracy, and precision [20,21,22,23,24].

A change in glucose levels, especially hypoglycemia, mediates autonomic activation and a change in the heart rate, sweating, and even consciousness level, which can be detected using wearables technologies [18,25,26]. An alteration in these variables may allow an indirect assessment of blood glucose levels [27,28,29]. To validate this claim, there is a need for a robust experimental design with appropriate types and numbers of participants and adequate data collection [30]. Very few longitudinal studies have assessed the utility of wearable sensors in relation to continuous glucose monitoring and the occurrence of hypoglycemia. This study differs from other previous studies as it recruited a large number of participants (i.e., 20 control, 20 with type 1 diabetes mellitus (T1DM), and 20 with type 2 diabetes mellitus (T2DM)) and used three wearables to acquire several different physiological outcomes simultaneously, and the data were collected under free-living conditions over four days.

We describe the key considerations whilst undertaking clinical studies using wearable devices to enable the acquisition of robust data to allow the development of glucose and hypoglycemia prediction models in patients with diabetes.

The contributions of this study are as follows:

1. Provide key considerations to enable a successful outcome for a clinical study using wearables.
2. Highlight the major challenges faced when undertaking a longitudinal study using wearables.
3. Recommend solutions to mitigate issues in the execution of a study using wearables.

## 2. General Considerations

There are general and ethical considerations that the research team must consider prior to conducting any clinical research activities involving participants.

### 2.1. Participants

Recruiting an adequate number of participants is key to establishing statistical power by satisfying the sample size representing the target population [31]. Recruitment begins by direct or indirect communication between the research team and potential participants by mailing, referrals, websites, and fliers [32,33]. Participant, research, and contextual factors can challenge the recruitment and retention of participants [33]. Poor and inefficient recruitment can increase the cost and affect the commitment of the research team with a potential for premature study termination with inadequate power to determine the primary outcome [34].

In this study, subjects with T1DM (n = 20) and T2DM (n = 20) were screened by their physician and referred to the research team for eligibility and consent. Healthy participants (n = 20) were enrolled through referrals and advertising at the approved sites. The participants had an average age of 36 years with 18 females and 42 males. Inclusion criteria were patients with type 1 or type 2 diabetes over the age of 18, treated with insulin. Exclusion criteria were: cardiac, liver, or renal dysfunction, vitamin B12 or folic acid deficiency, cancer, pregnancy or breastfeeding, and those who had undergone ocular surgery or trauma within the last 6 months, or had corneal pathology, or an allergy to eye drops.

### 2.2. Selection Criteria and Eligibility Screening

Eligibility criteria are set of specifications written in human-readable form highlighting the clinical characteristics of patients governing their entry into a medical trial [35,36]. They usually contain information about the enrollment conditions such as age, gender, diseases, physical disabilities, and medical conditions. Clinical trials also include exclusion criteria that describe factors that limit the eligibility of the participants. Such criteria should be decided by the medical team conducting the trials based on the objectives of the study and the target population. Table 1 shows the inclusion and exclusion criteria considered in the study.

### 2.3. Institutional Review Board

The Institutional Review Board (IRB) is comprised of individuals that oversee research protocols and ensure the adherence of investigators to regulations that protect human subjects participating in research [37]. Obtaining IRB approval is essential, but it can be frustrating and time-consuming for researchers that are not familiar with clinical research [38]. The primary investigator should provide a research protocol, patient information sheet, and consent forms to the IRB [39].

In the current study, the research was jointly reviewed and approved by three collaborating institutions. The approved documents included the main protocol containing the details of the objectives, background, inclusion and exclusion criteria, recruitment methods, procedures involved, statistical analysis, data management, withdrawal of participants, and risks to participants. Other approved documents included a diary log, an invitation email and flyer, an eligibility screening form, a consent form, a questionnaire, and a data collection sheet. The approved documents were in English and Arabic to meet the requirements of the local community.

### 2.4. Collected Data

Clinical studies and trials are conducted to collect data from eligible participants to answer specific research questions pertaining to the efficacy of a new treatment, educational approaches, screening methods, procedures, or a medical device [40]. Wearable devices can be used to conveniently acquire data on the vital signs of the participants, e.g., heart rate, temperature, respiration rate, activity level, and skin conductance [1]. In the current study, CGM devices and three wearables were used to collect interstitial glucose, heart rate, electrocardiogram, blood volume pulse, electrodermal activity, temperature, and acceleration.

## 3. Planning Your Experiment

This section highlights the steps towards a successful outcome to ensure that adequate data are captured from the wearable devices.

### 3.1. Glucose Monitoring

Continuous glucose levels are needed to establish their relationship with variable data acquired from wearable devices. Standard self-administered glucose measuring systems rely on a disposable test strip that reads the blood glucose from a drop of blood acquired through finger pricking. This method may cause discomfort and pain and provides only a limited number of data points. The CGM device measures the interstitial glucose continuously using a thin enzyme-coated subcutaneous sensor, which is comfortable and cost-effective compared to using blood test strips [20,23]. Although a CGM measures the interstitial glucose with an inherent time lag compared to blood glucose levels, it allows the continuous assessment of glucose alongside the wearables.

Currently, there are a number of CGM devices that are user-friendly, require no calibration, and conveniently acquire data. Two CGM devices were considered for the present study, namely the Dexcom G6 and FreeStyle Libre.

### 3.2. Wearable Devices

**Figure 1:**
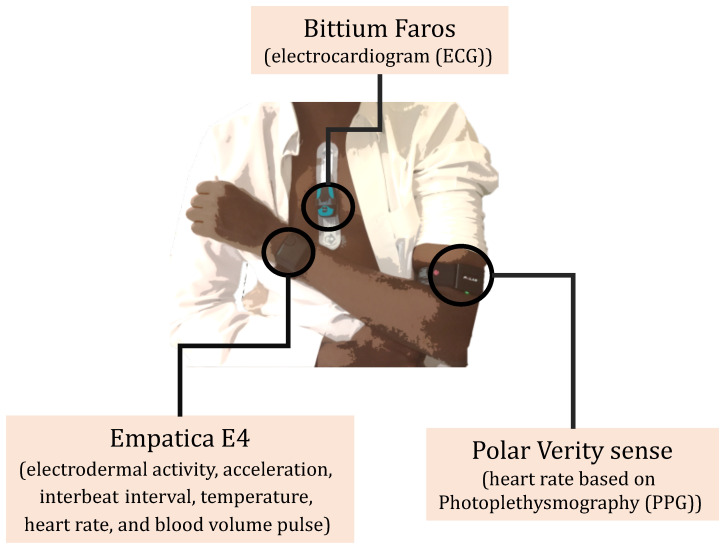
The three wearable devices used in this study.

When considering different wearables, one must take into account the modality, location, convenience, data storage, and battery. Three wearable devices were selected (Figure 1) to enable the collection of a range of physiological variables:

1. Empatica E4: A wristband that acquires electrodermal activity, acceleration, interbeat interval, temperature, heart rate, and blood volume pulse.
2. Bittium Faros: A chest wearable device that acquires a continuous electrocardiogram (ECG).
3. Polar Verity sense: An armband that uses photoplethysmography (PPG) sensors to detect the heart rate.

**Figure 2:**
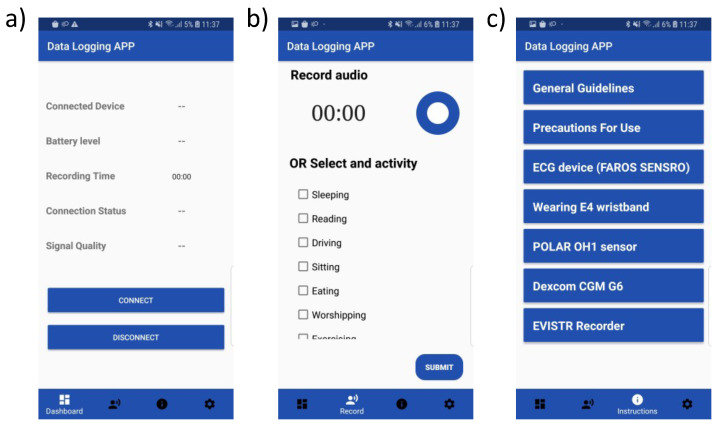
Screenshots of the developed App to interface with the armband wearable sensor and to register activities. (**a**) Main interface of the App to connect with the sensor. (**b**) Activity logging interface. (**c**) The general instructions and information interface.

These three devices vary in terms of their battery life and storage. The Bittium Faros can store data for up to 12 days without recharging. Empatica E4 can store data for approximately 67 h in offline mode and requires charging after 35 h. The Polar has limited built-in storage; hence, a phone App was developed to store the acquired data on dedicated study phones (Figure 2a). The research team produced a document with instructions on how to operate each device in relation to proper placement and data retrieval.

### 3.3. Tracking Logs

**Figure 3:**
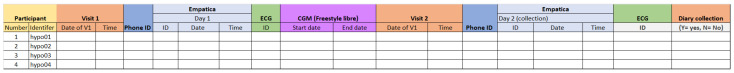
A snippet of the logging sheet.

A typical study using several wearables with many participants requires a common logging sheet shared among the team members to keep track of all the devices and the timings when they were given to the participants. A logging sheet is key to avoiding the misplacement of devices and mistakenly mixing the data of the participants. The logging sheet considered in the study contained an identifier for the participants along with details about the CGM, wearables, study phone, and time of visit (Figure 3).

Clear instructions were given to the medical team to log all the required details:

1. Write down the time when the wearables were given to the participant and when returned.
2. Write down the phone number (e.g., one or two to identify the CGM account).
3. Write down the device number at the back of the watch under “E4” (e.g., AXXBXX) to identify which device was given to the participant.
4. Create a main unique folder name for each participant.

### 3.4. Task Distribution and Resource Allocation

**Figure 4:**
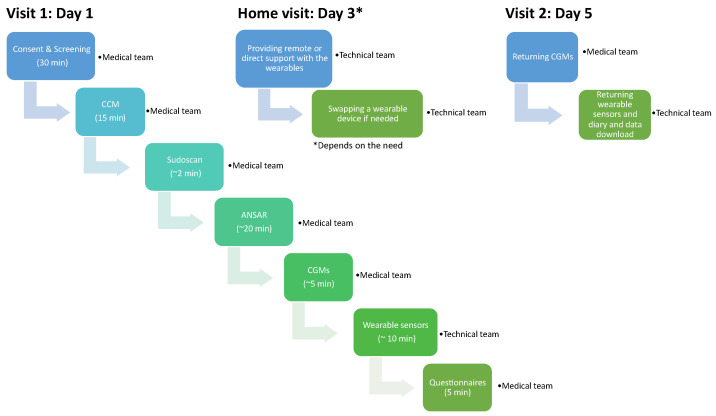
An overview of the task distribution during the 4-day trial.

All assigned tasks should be clear and appropriate with respect to the team members’ expertise. In this study, the principle investigators monitored and oversaw the study, medical investigators screened and recruited the participants, and the technical team helped with the deployment of the wearable devices and the acquisition of the data (Figure 4).

### 3.5. Daily Activity Logging

**Figure 5:**
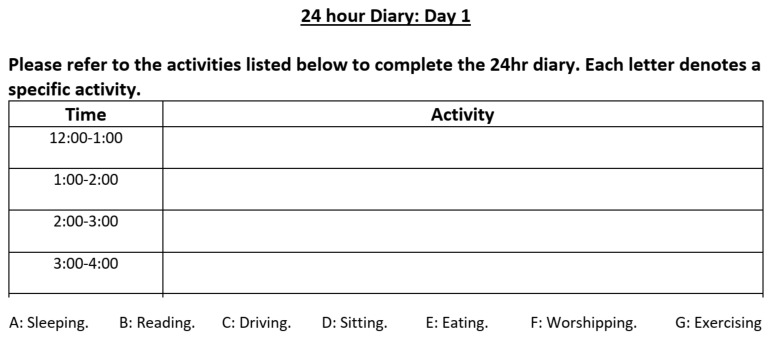
A snippet of the daily diary used by the participants to record their hourly activities.

Activity recognition using self-annotated data or sensory readings (e.g., accelerometer) from wearables is useful in healthcare applications [41,42,43]. Being aware of the current physical activity of each participant is key to interpreting changes in blood glucose levels and, especially, the development of hypoglycemia [44,45]. Previous studies have investigated the effects of physical activity on blood glucose in patients with diabetes [44,46]. To collect the daily activities of the participants, they were asked to log their activities using a sheet (Figure 5) or an App (Figure 2b) with a list of possible activities. The participants were instructed to record the duration of their activities by marking the beginning of a long activity (>5 min) and annotating it while ignoring a short activity (<5 min).

### 3.6. Hardware Troubleshooting

Using multiple devices (e.g., wearables and CGMs) and their Apps can lead to issues in data collection that require troubleshooting to resume the normal intended mode of operation. It is important to compile a list of all possible malfunctioning scenarios that the participant may face with the wearables along with steps to troubleshoot them to ensure robust data collection. Instructions on the proper usage of wearables were also included in the developed App (Figure 2c).

### 3.7. Study Protocol and Pilot Experiments

A successful study should include a detailed and clear protocol highlighting the procedures of the study. Prior to recruiting participants, it is essential to test the protocol and devices to evaluate their feasibility, accuracy, and efficiency [31]. Pilot experiments help to identify technical issues with the wearables, bugs with the software (e.g., developed App), and difficulties the participants might face in the actual experiments. In this study, individual team members tested the CGM and wearables for four days to identify potential issues, which were addressed prior to commencing the study. For example, armband or wristband wearables might cause irritation or discomfort due to prolonged usage; hence, a recommendation was given to change the location or adjust the wearable devices during the study.

## 4. Neuropathy Assessment

### 4.1. Corneal Confocal Microscopy

**Figure 6:**
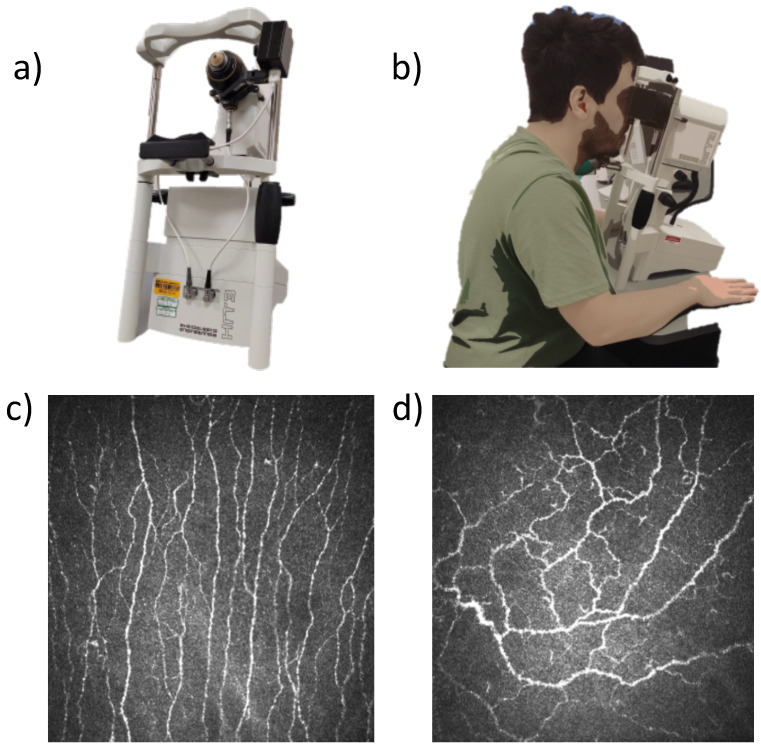
Corneal confocal microscopy screening test. (**a**) The CCM device. (**b**) Demonstration of the test. (**c**) Central corneal sub-basal nerve plexus. (**d**) Inferior whorl.

Corneal confocal microscopy (CCM) is a rapid, non-invasive, and well-tolerated technique to detect and quantify neurodegeneration in adults and children with diabetes [47,48]. CCM was undertaken using the Heidelberg Retina Tomograph Cornea Module (Figure 6a, Heidelberg Engineering, Heidelberg, Germany). Both eyes were anesthetized with two drops of Bausch & Lomb Minims (Oxybuprocaine hydrochloride 0.4% *w*/*v*). A drop of hypotears gel (Carbomer 0.2% eye gel) was placed on the tip of the objective lens, and a sterile disposable TomoCap was placed over the lens, allowing optical coupling of the objective lens to the cornea. Six images were selected from the sub-basal nerve plexus in the central cornea (Figure 6c) and the inferior whorl (Figure 6d) to manually quantify corneal nerve fiber density (fibers/mm^2^), corneal nerve branch density (branches/mm^2^), corneal nerve fiber length (mm/mm^2^), corneal nerve fiber tortuosity, and inferior whorl length using CCMetrics. The investigator was blind to the study group when performing CCM and analyzing CCM images.

### 4.2. Sudomotor Function (Sudoscan)

**Figure 7:**
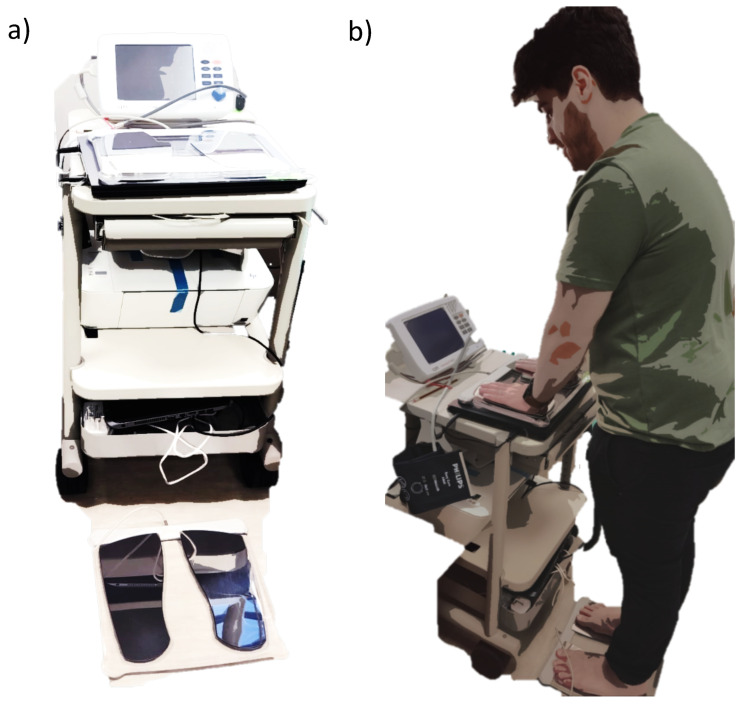
The Sudoscan device used to perform the sudomotor test. (**a**) The device. (**b**) Demonstration of the test.

Sudoscan (Impeto Medical, Paris, France) is a noninvasive sudomotor test that allows the measurement of sweat gland function and, hence, peripheral sympathetic integrity (Figure 7a). The Sudoscan test is based on the electrochemical reaction between the chloride ions in sweat and stainless-steel plate electrodes, where the subject’s hands and feet are placed [49]. The device generates a low-voltage current (<4 V) that attracts the chloride ions from the sweat glands on the palms and soles to measure electrical conductance (ECS) in both the hands and feet. Sudomotor dysfunction is evaluated according to the ESC measured on the feet: >60 μS = no dysfunction; 60–40 μS = moderate dysfunction; <40 μS = severe dysfunction. During the test, subjects were required to place their hands and feet on the electrodes and to stand still for 2–3 min (Figure 7b). The device produces average ESC results for the hands and feet [49].

### 4.3. Cardiac Autonomic Function (ANSAR)

**Figure 8:**
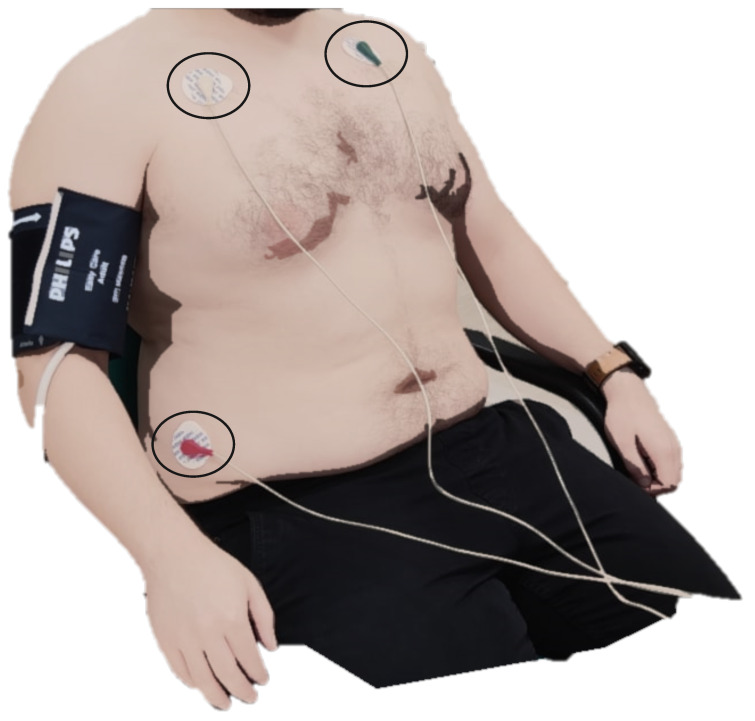
The placement of the 3 ECG electrodes on the chest highlighted with circles.

Cardiac autonomic profiling was performed using ANX-3.0 (ANSAR Medical Technologies, Inc., Philadelphia, PA, USA). Subjects were instructed to refrain from smoking and coffee consumption before the test. The test was performed while the subject was seated; a blood pressure cuff was placed on the left arm, and three ECG leads were placed below the clavicle, each approximately 1.5–2 inches from the sternum and the third at the base of the rib cage (Figure 8).

The test is based on six different breathing patterns (normal, deep breathing, and Valsalva) and blood pressure measurements while sitting and standing. The ANX test assesses sympathetic and parasympathetic activity, as well as sympathovagal balance to provide a measure of cardiac autonomic neuropathy.

## 5. Data Handling and Processing

The wearable devices acquire data of different modalities and at different rates and are susceptible to noise and motion artifacts. Preprocessing of the data is required for the machine learning application.

### 5.1. Data Upload and Backup

A clear set of instructions was required to retrieve the data from the different wearable devices. After collecting a participant’s data, it was imperative to create multiple secure backups in different storage devices and on the cloud to avoid data loss.

### 5.2. Preprocessing

Preprocessing of the data from the wearables was needed to accommodate different sampling rates, noise, and missing values. In this study, three wearables to collect data of different modalities sampled at various frequencies were used. For example, the ECG signal was acquired at 250 Hz, while acceleration on the Empatica was at 34 Hz. Hence, resampling was required to ensure that all the data matched. Upsampling using linear interpolation and downsampling were performed on the wearables and CGM data to unify the sampling frequency at 125 Hz.

Readings acquired from sensors and wearable devices are susceptible to motion artifact, temperature change, and baseline drifting, for which filtering techniques are needed to counter or mitigate their effects. For the ECG signal, the filtering technique proposed by Gamboa and implemented by the NeuroKit2 python library was used [50,51]. For the PPG signal, the approach proposed by Elgendi et al. was used, and for the acceleration, skin temperature, and electrodermal activity signals, a moving average filter was implemented for each with a window size equal to half of their respective sampling frequencies [52].

When dealing with data containing large amounts of noise, it is useful to establish a quality index to identify the usable and noise-free data. For example, when dealing with the ECG signal, the team considered the MIT-BIH noise stress dataset and tested five different ECG quality index methods [53]. These methods were based on detecting QRS point differences (average QRS) [51], correlation between beats and average beat representation, the Signal Quality Index (SQI) [54], and the 1D convolutional neural network (CNN) model. Based on the results (Table 2), the 1D CNN achieved the best outcomes and was, thus, considered as the quality index.

Missing data from wearables and CGM occurred due to many factors such as running out of battery charge, accidentally turning off the device, random malfunction, and forgetting to scan regularly. When using more than one device to collect data from the participants, there is a high potential for missing data in one of the devices, and the regions of missing data collected by a wearable device are unusable. Hence, to achieve consistency, these regions should be discarded from all the other devices being used in parallel when the contemporaneous data of all are needed in an application. However, when the missing data from a wearable device are many (>60% of a session), then that specific sensor reading should be dropped. When the reference device (e.g., CGM) values are largely missing, then that participant’s data are unusable. The research team members handling the data analysis should decide what to keep or discard depending on the amount of missing data and their objectives.

## 6. Challenges

This section highlights the many challenges faced during this study involving the collection of multiple datasets from participants using wearables. Some challenges can be avoided, whilst others can only be mitigated.

### 6.1. Challenges Related to Approvals

Approvals from the institutional review board are essential in any clinical research involving human subjects to ensure the protection of the rights and welfare of human research subjects involved in the study. However, approval can take time, thus delaying the start of a study, or may require a pause mid-study if an issue is identified that may potentially cause harm to the participants, or there is a delay in the submission of the continuation report. This study was a collaboration between Hamad Medical Cooperation, Qatar University, and Weill Cornell Medicine Qatar; therefore, approvals to start the study and to make amendments were a challenge.

### 6.2. Challenges Related to Recruitment

There are many issues and challenges associated with the recruitment and participation of subjects in a clinical study [32,33,55]. Eligible subjects were referred by their physician to the research team to enable a detailed explanation of the study, following which some subjects refused to participate due to:

1. No direct or obvious benefits to the subjects.
2. Length of the visit. The first research visit included: consenting, the CCM test, Sudoscan, the ANSAR test, providing wearables, and explaining how to use each device (1 to 2 h for each subject).
3. The number of wearable devices discouraged participants, especially females.
4. The need to log activities for four days.
5. The study was conducted during the COVID-19 pandemic, making subjects more hesitant to come to the hospital.

A study logistics issue that was hindering recruitment was the number of wearable device sets available to the research team (i.e., five sets), which limited recruitment to no more than five subjects per week. Additionally, due to technical issues, the number of sets available was at times reduced to three sets, further reducing recruitment to three subjects per week.

### 6.3. Challenges Related to Screening

Difficulties in screening depend on the following:

1. Access to clinical data on electronic medical record systems (Cerner).
2. Inclusion and exclusion criteria.

In this study, screening was performed by the physician principle investigator and there was no difficulty in accessing the database. Some of the inclusion and exclusion criteria made it more difficult to find eligible subjects.

There were challenges related to the use of three tests used to define neuropathy (i.e., CCM, ANSAR, and Sudoscan):

1. Difficulty for the participants to sit still for the duration of the tests and to follow instructions properly.
2. Eye movement during the CCM test. It was hard for the participants to avoid moving their eyes and blinking during the test, increasing the time needed for the examination.
3. Length of the test. For example, on average, CCM took 10–15 min for each participant.
4. Valsalva maneuver in the ANSAR test: it was somewhat difficult for some of the participants to hold their breath for 15 s.

### 6.4. Challenges Related to Subject’s Compliance

**Figure 9:**
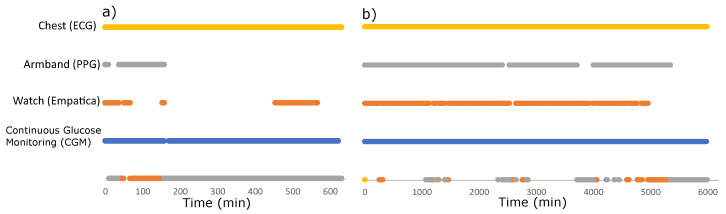
Data availability for the CGM and three wearables acquired from two participants demonstrating the discrepancy in the amount of data collected from each participant. (**a**) Worst case. (**b**) Best case. Note: the points on the time axis represent missing data.

Even after referral and confirmation, several participants did not attend the hospital to participate. Furthermore, 6/60 participants did not complete the 4-day trial and returned the wearables early, creating considerable discrepancy in the amount of data collected between participants (Figure 9).

The commitment of the participants to continuously collect data and log their activity was challenging [56]. In studies using wearable devices, considerable commitment is required from the participants to adhere to the protocol for collecting continuous and contemporaneous data with steps to limit the amount of missing data [57]. Some might face difficulties in following the instructions to record the data properly. For example, some of the old participants in this study faced some technical difficulties in operating the wearable devices. Timely communication amongst the team members and participants was essential in mitigating the loss of data. Continuous communication and support were provided by the technical team to guide and help the participants and to remind them to check whether the wearables were working properly. Upon completing the study, the wearable devices were returned to the research team with a visit to the hospital. However, some participants were delayed due to work, which created delays in recruiting new participants, as this affected the data retrieval and access to the wearables. Device malfunction may also create delays, and there should be rapid access to extra wearables. A lack of wearable units and the long shipping time also created delays and caused a bottleneck in the number of participants recruited.

### 6.5. Device-Related Challenges

The research team faced significant challenges with the configuration and interaction with the data collection with the Dexcom CGM device, which required a change to the Freestyle Libra and, hence, a delay in starting the study due to a change in the protocol and IRB approval.

The selection of wearable devices for longitudinal studies can be challenging. The design and structure of a wearable device need to be both robust to withstand normal daily living conditions and comfortable for the user to be worn over extended periods of time. Patch-based wearables, such as the ECG, require direct skin contact and access to sanitizing and shaving kits before application. In this study, a few participants faced issues with the patch of the CGM and ECG, as they came off accidentally during sports or sleeping. There may also be issues associated with wearing devices for 4 days, which may cause discomfort or irritation of the skin. The participants were instructed to either re-adjust the tightness of the straps, change the location of the wearable device, or take a short break from wearing it.

The battery life of wearable devices is critical in longitudinal studies collecting continuous data. The wearable device should require no to minimum troubleshooting and infrequent charging. Wearable devices that need to be charged often will create more missing data. Of the wearables deployed, the ECG device lasted the longest for up to 12 days without the need for charging, and indeed, the data availability of the ECG was the highest of all the wearables (Figure 9). The armband PPG battery lasted 18 h and had the lowest data availability. To reduce missing data, the participants received an extra unit to swap whilst charging and a reminder was added in the developed App to let the participant know when to swap and charge the device.

## 7. Concluding Remarks

The outcome of a clinical study using wearables can be challenging due to multiple factors. This article was limited to highlighting the challenges and to providing recommendations to address issues affecting the validity of collecting data from wearables. We identified the usual issues pertaining to delays in IRB approval and screening and identifying eligible participants. However, in addition, issues around continuous data collection from wearables require extra considerations and specific solutions.

After finalizing the set of wearables to be used, the research team should produce manuals with clear instructions on how to use the wearable devices and how to retrieve the data. Long-term use of wearables may cause discomfort and irritation to the skin. The limit of the number of wearables requires effective cleaning and maintenance with the anticipation of malfunctioning and missing units.

Prior to starting the clinical study, the research team should test all the devices to identify issues in the hardware or bugs in the software. The research team should also have strategies to reduce the burden on the participants, e.g., provide training upon using the wearables with continuous help and support during the period of data acquisition. The research team may consider developing their own App to provide a basic troubleshooting guide, assist in storing wearable sensor data, remind the participants to upload data in order to reduce missing data, and annotate data in relation to the activity.

The research team may also need to explore various filtering and data-processing techniques to optimize data collection. Apart from successful data collection, future research utilizing wearables to enable glucose monitoring should focus on improving the accuracy, reliability, and convenience, as well as deploying AI and other emerging technologies to provide personalized and effective diabetes management strategies.

## Figures and Tables

**Table 1:** Inclusion and exclusion criteria.

Inclusion Criteria	Exclusion Criteria
Adults aged 18–65 years old	Failure to provide consent
Arabic and English speakers	Pregnancy
Physically able to undergo the screening tests	With a specific medical history and on medication for acute illnesses

**Table 2:** Noise classification performance of different signal quality index calculation methods and CNN.

Method	F1	Precision	Recall	Accuracy	Inference Time (100 Samples)
Correlation	75.18	60.72	98.70	90.20	2.11 s
Average QRS [51]	91.93	98.97	85.82	97.73	5.13 s
SQI [54]	87.65	80.25	86.54	95.91	0.92 s
CNN	96.22	96.63	95.83	98.87	0.14 s

## Data Availability

Not applicable.

## Abbreviations

The following abbreviations are used in this manuscript:

CGM	Continuous glucose monitoring
T1DM	Type 1 diabetes mellitus
T2DM	Type 2 diabetes mellitus
IRB	Institutional review board
ECG	Electrocardiogram
PPG	Photoplethysmography
CCM	Corneal confocal microscopy
Sudoscan	Sudomotor function
ECS	Electrical conductance
ANSAR	Cardiac autonomic function
SQI	Signal Quality Index
1D CNN	One-dimensional convolutional neural network
